# Intergenerational Impact of Violence Exposure: Emotional-Behavioural and School Difficulties in Children Aged 5–17

**DOI:** 10.3389/fpsyt.2021.771834

**Published:** 2022-01-04

**Authors:** Ladan Hashemi, Janet Fanslow, Pauline Gulliver, Tracey McIntosh

**Affiliations:** ^1^Department of Social and Community Health, Faculty of Medical and Health Sciences, School of Population Health, University of Auckland, Auckland, New Zealand; ^2^Māori Studies and Pacific Studies, Faculty of Arts, University of Auckland, Auckland, New Zealand

**Keywords:** intergenerational impact, parental violence exposure, emotional-behavioural difficulties, school difficulties, child's outcomes, New Zealand

## Abstract

**Background and Objectives:** The intergenerational impacts of parental exposure to violence during childhood and adulthood have largely been investigated separately. This limits our understanding of how cumulative violence exposure over a lifespan elevates the risk of subsequent generation's maladjustment. To address this, we examined if parental exposure to violence during childhood and during adulthood was associated with increased emotional-behavioural and school difficulties among the children of these parents. Further, we examined if parental exposure to cumulative violence increased the odds of their children experiencing difficulties.

**Participants and Setting:** 705 participants (354 mothers and 351 fathers) from the 2019 New Zealand Family Violence Survey, a population-based study conducted in New Zealand between March 2017 and March 2019.

**Methods:** Multivariable logistic regressions were conducted to ascertain the impact of parental exposure to violence on children's outcomes after adjustment for sociodemographic characteristics. The impact of parental cumulative violence exposure on children's outcomes was also explored.

**Results:** Findings indicated that children of parents who had histories of exposure to violence during childhood were at increased risk for experiencing emotional-behavioural or school difficulties. However, where parents reported a history of childhood abuse but not adult experience of violence, their children had similar odds of experiencing difficulties as the children of parents who had not been exposed to any violence in their lifetime. Children of parents who had been exposed to violence only during adulthood were at higher risk of experiencing emotional-behavioural difficulties compared with children of parents with no violence exposure. Children of parents with histories of exposure to violence during both childhood and adulthood had the highest prevalence of experiencing emotional/behavioural and school difficulties.

**Conclusion:** These findings highlight the intergenerational impacts of violence exposure and the complex intersections between parents' and children's life experiences. Our findings suggest the need for violence prevention initiatives to foster the development of safe, stable and nurturing relationships and to expand services for parents already exposed to violence to build resilience and to break the inter-generational cycle of disadvantage.

## Introduction

Intergenerational impacts of violence exposure refer to how parental exposure to violence during childhood or adulthood affects their children. Research in this area has often focussed on childhood exposure as a risk factor for later risk of violence perpetration (often called “intergenerational transmission” of violence). However, there is also a need to investigate other intergenerational impacts that may occur. Research has shown that the consequences of childhood exposure to violence affects people's social, economic, and physical chances throughout the lifespan (1), and may lead to multiple and complex experiences of disadvantage, including higher likelihood of repeat victimisation and exposure to violence in adulthood (2, 3).

Additionally, there is evidence that childhood exposure to violence can create negative outcomes for successive generations (4). For example, it has been documented that parental exposure to violence during childhood has implications for their later parenting practices (5). Similarly, parent's exposure to violence during adulthood is associated with adverse outcomes for their children. For example, intimate partner violence (IPV), one of the most common forms of violence, has negative consequences not only for individuals subjected to violence but also for children of those affected (6). This includes negative effects on children's intellectual (7) emotional, behavioural and social development (6), as well as on their academic performance (8). Most studies exploring intergenerational impacts of violence have explored the effects of parent's exposure to IPV, but fewer have examined how parent's exposure to violence by non-partners impacts on their children. As with IPV, non-partner violence can include physical or sexual violence, and can be perpetrated by a range of individuals, including other family members (non-partners), acquaintances or strangers.

Further, the intergenerational impacts of parental exposure to violence during childhood and adulthood have largely been investigated separately (9). This has limited our understanding of how cumulative violence exposure over a lifespan elevates the risk of subsequent generation's maladjustment. The limited research that has been conducted on intergenerational impacts suggests that parental exposure to violence as a child along with IPV exposure as an adult exacerbates the likelihood of negative outcomes for subsequent children. Another gap in the literature is that the majority of research on adult exposure to violence has either focused on the effects of IPV on the mothers as victims and fathers as perpetrators and the outcomes for their children (9). At present, there is a paucity of evidence exploring how fathers' experience of violence impacts on their children. While both men and women can be victims as well as perpetrators, differences between men's and women's experiences of violence have been noted. For example, women are more likely to experience physical and mental health problems as the consequence of violence exposure than men (10, 11). Male-perpetrated IPV has also been shown to be more injurious for women and result in more severe short and long term sequalae (12). Consequently, women are also more likely to be killed as a result of IPV (13, 14).

The field would also benefit from additional exploration of intergenerational impacts beyond consideration of children's externalising and internalising problems, two of the most consistently documented factors related to parental exposure to violence (5, 14, 15). More specifically, the impact of parental exposure to violence on children's educational outcomes is understudied and less understood (5, 16, 17). Finally, critical reviews have called attention to methodological limitations of research in this field (12), such as the fact that most studies on violence exposure are drawn from service data (e.g., from clients accessing mental health services or child protective agency records). These samples represent only a proportion of violence exposure, i.e., include only cases that comes to the attention of the authorities (13), so their results may not be generalizable to the whole population or to unreported cases of abuse. Data from population-based samples are required to further validate these findings.

### Current Study

Using data from a large population-based study in New Zealand we examine if parental exposure to violence during childhood and during adulthood (violence by an intimate partner and/or a non-partner) is associated with increased levels of emotional-behavioural and school difficulties among the children of these parents. Further, we examine if parental exposure to cumulative violence increases the odds of their children experiencing difficulties.

## Materials and Methods

The data reported are from the 2019 New Zealand Family Violence Survey/He Koiora Matapopore, a population based study conducted in three regions (Waikato, Northland and Auckland) in New Zealand between March 2017 and March 2019. Full details of the study methods are published elsewhere (15) but are summarised briefly here. Eligibility requirements for participants were: age 16 years and over, speaking conversational English, sleeping in the property at least four nights a week on average and living at the property for at least 1 month prior to data collection. Both women and men were recruited for the study.

### Sampling Method

Meshblocks (the smallest geographical unit used for census surveys) were selected by Stats NZ. Within each meshblock, a random starting point was identified and every second and sixth house within the meshblock was selected. Non-residential and short-term residential properties, rest homes and retirement villages were excluded. Specific meshblocks were allocated to each gender for safety reasons. In addition, only one randomly selected person per household could participate in the study.

### Data Collection

Data was collected through face-to-face interviews using the WHO Multi-Country Study on Violence Against Women (VAW) questionnaire (16). The instrument was adapted to include men and was pre-tested with a convenience sample before the actual data collection started. Questions on childhood exposure to violence was taken from the Adverse Childhood Experience study questions used by the USA Centres for Disease Control and Prevention (17). Comprehensive training of all interviewers was conducted to ensure valid data collection and the safety of interviewers and respondents. For quality assurance purposes regular meetings, audits and reviews of completed interviews were conducted. Interviews were conducted privately with no one aged 2 years or over present. All respondents provided written consent prior to interview.

### Study Sample

The final sample size for the 2019 New Zealand family violence study was 2,887 and consisted of 1,423 men and 1,464 women who completed interviews. Those who agreed to participate represented over 60% of eligible individuals (63.7% women, 61.3% men).

### Representativeness

The ethnicity, marital status, average personal income, and deprivation level distribution of the original sample were closely comparable to the general population, however, the sample was under-represented for younger respondents (ages 16–29) and slightly over-represented for those over 60 years of age (15).

**Figure 1:**
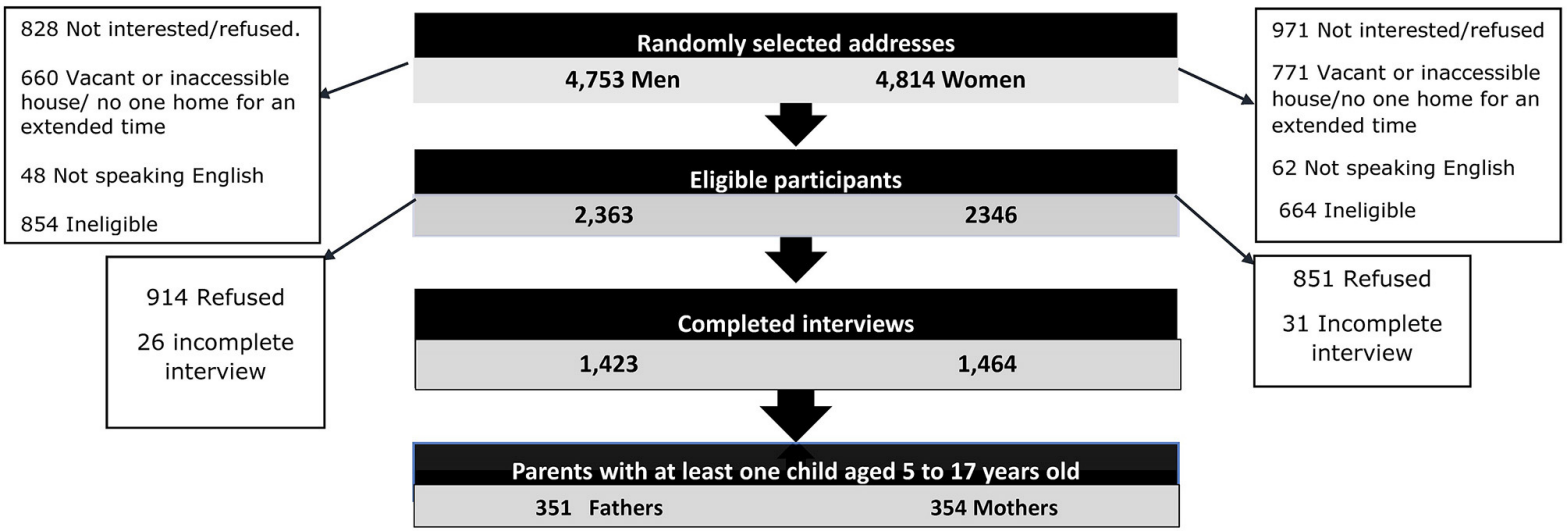
Flow diagram of included participants.

This study uses data from 354 women (mean age = 42.0 years, SD = 7.0) and 351 men (mean age = 45.1 years, SD 7.4) who had at least one child aged 5–17 years old at the time of interview. Figure 1 documents households approached, contacted and the recruitment outcomes at the individual level. Demographic characteristics of the study sample are presented in **Table 2**.

### Measures and Variables

Information on children's emotional-behavioural and school difficulties were collected from the parent (respondent) only. Respondents were instructed to consider their children aged 5–17 years when answering questions concerning children's outcomes.

### Main Outcomes of Interest

The main outcomes of interest for the current study were child emotional-behavioural and school difficulties reported by their parents (respondents). To measure emotional/behavioural difficulties, four items were used: *child emotional difficulties* (two items): having nightmares; being timid or withdrawn; and *child behavioural difficulties* (two items): being aggressive; running away from home. A binary variable was created to measure child experience of any emotional-behavioural difficulties (any / none). Two items were used to measure *school difficulties*: being truant; being suspended from school. A binary variable was created to measure child's school difficulties (any/none). *Don't know* or *can't remember* were treated as missing data. Exact questions wording and response options are provided in Table 1.

**Table 1:** Definition of food security, children's emotional-behavioural and school difficulties, and parental violence exposure during childhood and adulthood, the 2019 family violence study.

**Variable**	**Definition**
Food security status	Do you ever worry about not having enough money to buy food? We scored responses of “never” as 0 and all other responses (Occasionally/Sometimes/Often/All the time) as 1.
**Children's emotional-behavioural difficulties**
Having nightmare	Do any of these children (aged 5–17 years) have frequent nightmares? (yes/no)
Being timid or withdrawn	Are any of these children (aged 5–17 years) very timid or withdrawn? (yes/no)
Being aggressive	Are any of them aggressive (aged 5–17 years) with you or other children? (yes/no)
Running away from home	Have any of these children (aged 5–17 years), ever run away from home? (yes/no)
**Children's school difficulties**
Being truant from school	Have any of your children (aged 5–17 years) ever been truant from school? (yes/no)
Being suspended from school	Have any of your children (aged 5–17 years) ever been suspended from school? (yes/no)
**Violence exposure during childhood**
Emotional abuse	While you were growing up, in your first 18 years of life: did a parent or adult in your home ever swear at you, insult you, or put you down? (yes/no)
Physical abuse	Before age 18, did a parent or adult in your home ever hit, beat, kick, or physically hurt you in any way? Do not include smacking (yes/no)
Sexual abuse	Before the age of 15, do you remember if anyone ever touched you sexually, or made you do something sexual that you didn't want to do? (yes/no)
Intimate partner violence witnessing	While you were growing up during your first 18 years of life, was your mother or step mother ever slapped, hit, kicked, punched or beaten up? (yes/no)
**Violence exposure during adulthood**
Physical or sexual Intimate Partner Violence (IPV)	Respondents were classified as exposed to physical or sexual violence perpetrated by an intimate partner if they responded affirmatively to at least one of the following questions on physical or sexual IPV. Physical IPV: has any partner ever (a) slapped you or thrown something at you that could hurt you?, (b) pushed or shoved you or pulled your hair?, (c) hit you with their fist or with something else that could hurt you? (d) kicked, dragged or beaten you up? (e) choked or burnt you on purpose?, (f) threatened to use or actually used a gun, knife, or other weapon against you. Sexual IPV: has any partner ever (a) physically forced to have sexual intercourse when you did not want to?, (b) having sexual intercourse because she was afraid of what her partner might do or being forced to do something sexual that she found degrading or humiliating.
Physical or sexual non-partner violence	Respondents were classified as exposed to physical or sexual violence perpetrated by a non-partner if they responded affirmatively to at least one of the following questions on physical or sexual violence. Physical non-partner abuse: since the age of 15, has anyone (other than your partner) ever hit, beaten or done anything else to hurt you physically? Sexual non-partner abuse: since the age of 15, has anyone (other than your partner) ever forced you to have sex or to perform a sexual act when you did not want to (by threatening you, holding you down or putting you in a situation that you could not say no)?

### Exposures of Interest

The main exposure variables in the current investigation were parental exposure to violence during childhood and adulthood. In this study, we defined *exposure to violence during childhood* as being directly subject to violence or being exposed to violence against their mother/stepmother. Based on this, exposure to four types of violence during childhood were included: direct experience of psychological abuse, physical abuse, sexual abuse and witnessing IPV against mother or step-mother. A binary variable called CAN (child abuse and neglect) was created to measure any violence exposure during childhood vs. none.

*Exposure to violence during adulthood* included exposure to two types of violence: physical or sexual violence by an intimate partner (IPV) and physical and or sexual violence by a non-partner. A binary variable was created to measure any physical or sexual violence exposure during adulthood vs. none (Table 1).

*Cumulative violence exposure* was defined by combining the two derived binary variables (exposure to at least one type of violence during childhood and exposure to at least one type of violence during adulthood). Four groups were defined: those with no violence exposure during childhood and adulthood, those with violence exposure only during childhood, those with violence exposure only during adulthood, and those with violence exposure during both childhood and adulthood.

### Socio-demographic Factors

Sociodemographic variables were used to explore prevalence rates of reported child emotional-behavioural and school difficulties among sub-populations and as potential confounders in the multivariable analyses (18–21). These variables included parental ethnicity (European, Māori, Pacific, Asian, MELAA [Middle East or Latin American or African]) and food security status (secure, insecure). Since there were too few MELAA respondents who had children aged 5–17 (*n* = 4 women and *n* = 7 men), this ethnic group was dropped from the analyses. The definition for food security status is presented in Table 1.

### Analytic Procedure

Analyses were conducted with Stata 15 SE (22). In all analyses, the complex sampling design has been allowed for through use of the Survey Data Analysis programs in Stata/SE, which allows for stratification by sample location (region), clustering by PSUs, and weighting of data to account for the number of eligible participants in each household.

Descriptive statistics including frequency and weighted percentages were reported for the outcome variables (parental report of child's emotional-behavioural and school difficulties) and for each parental socio-demographic and violence exposure variable for the study sample (Tables 2, 3). Prevalence rates (weighted percentages) of the outcome variables were also estimated across socio-demographic sub-groups (gender, ethnicity, food security status) and violence exposure variables (Table 2). Univariate logistic regression was used to investigate association between (a) parental socio-demographic characteristics and reports of child outcomes, (b) parental report of violence exposure during childhood and adulthood and child outcomes, and (c) parental report of violence exposure during their childhood and their adulthood. Odds ratios are reported with 95% confidence intervals.

**Table 2:** Child's emotional-behavioural and school difficulties by parental socio-demographic characteristics and parental violence exposure, from the 2019 New Zealand Family Violence Study.

		**Child's emotional-behavioural difficulties**					**Child's school difficulties**		
	**Study**	**Nightmare**	**Timid**	**Aggressive**	**Run away**	**At least**	**Being truant**	**Being suspended**	**At least one**
	**sample**				**from home**	**one behavioural-**	**from school**	**from school**	**school difficulty**
	***n* (W%)**					**emotional difficulty**			
Study sample *n* (W%)		57 (7.8)	80 (11.9)	67 (9.6)	18 (2.8)	171 (24.9)	44 (7.5)	26 (4.3)	54 (9.1)
Relationship with child									
Father (ref, W%)	351 (49.6)	4.3	11.9	7.0	3.1	19.8	6.5	3.9	7.5
Mother (W%)	354 (50.3)	11.2	12	12.2	2.4	29.8	8.4	4.8	10.6
OR		**2.5 (1.39–4.52)**	1.00 (0.60–1.70)	**1.84 (1.02–3.33)**	0.75 (0.27–2.03)	**1.71 (1.16–2.53)**	1.31 (0.66–2.59)	1.24 (0.50–3.05)	1.45 (0.78–2.69)
Ethnicity									
European (ref, w%)	440 (59.1)	8.2	13.4	9.8	2.7	26.4	6.0	3.1	7.9
Maori (w%)	91 (14.7)	10.7	9.2	15.7	5.0	29.7	16.0	8.3	16.7
OR		1.34 (0.63–2.83)	0.65 (0.29–1.48)	1.70 (0.81–3.5)	1.93 (0.55–6.73)	1.17 (0.66–2.07)	**2.97 (1.30–6.81)**	2.82 (0.97–8.20)	**2.33 (1.07–5.09)**
Pasifika (W%)	53 (9.4)	6.5	11.7	7.7	3.8	28.9	16.7	12.8	19.2
OR		0.77 (0.14–4.25)	0.86 (0.27–2.71)	0.76 (0.27–2.13)	1.45 (0.28–7.55)	1.13 (0.51–2.49)	**3.13 (1.25–7.80)**	**4.60 (1.56–13.5)**	**2.77 (1.21–6.35)**
Asian (W%)	109 (17.7)	4.4	8.8	4.4	0.7	12.6	0.7	0.7	1.4
OR		0.51 (0.21–1.25)	0.63 (0.27–1.43)	0.42 (0.13–1.41)	0.26 (0.33–2.08)	**0.40 (0.20–0.78)**	**0.11 (0.01–0.88)**	0.23 (0.3–1.83)	**0.17 (0.04–0.74)**
Food security									
Secure (ref,W%)	527 (74.0)	7.0	9.4	7.6	2.3	21.2	5.5	2.8	6.7
Insecure (W%)	177 (26.0)	10.3	19.1	15.3	4.1	35.5	13.1	8.7	15.9
OR		1.53 (0.79–2.96)	**2.27 (1.34–3.85)**	**2.18 (1.21–3.94)**	1.8 (0.65–5.30)	**2.04 (1.34–3.13)**	**2.59 (1.35–4.96)**	**3.36 (1.51–7.48)**	**2.63 (1.45–4.77)**
**Parental exposure to violence during childhood**									
Psychologically abused as a child	229 (34.4)								
Yes (W%)		8.4	15.8	12.2	4.6	31.0	12.0	5.2	12.8
No (ref, W%)		7.4	10.0	8.3	1.8	21.7	5.2	3.9	7.2
OR		1.16 (0.62–2.17)	**1.70 (1.03–2.81)**	1.54 (0.87–2.70)	2.54 (0.89–7.25)	**1.61 (1.1–2.37)**	**2.52 (1.28–4.93)**	1.37 (0.55–3.38)	**1.88 (1.03–3.44)**
Physically abused as a child	127 (20.8)								
Yes (W%)		7.5	11.6	15.0	7.6	27.8	14.7	9.8	15.9
No (ref, W%)		7.8	12.1	8.2	1.5	24.1	5.6	2.9	7.4
OR		0.96 (0.47–1.93)	0.96 (0.51–1.78)	**1.96 (1.06–3.63)**	**5.36 (1.87–15.34)**	1.21 (0.75–1.96)	**2.87 (1.46–5.64)**	**3.64 (1.61–8.2)**	**2.37 (1.26–4.48)**
Sexually abused as a child	125 (18.4)								
Yes (W%)		11.8	17.2	21.4	2.8	41.2	14.7	6.2	15.3
No (ref, W%)		6.5	10.6	7.2	2.3	20.5	5.4	3.6	7.3
OR		**1.91 (1.01–3.62)**	1.75 (0.96–3.17)	**3.52 (1.90–6.52)**	1.19 (0.36–3.88)	**2.73 (1.75–4.25)**	**2.99 (1.50–5.94)**	1.77 (0.66–4.73)	**2.27 (1.18–4.37)**
IPV witnessing	121 (17.4)								
Yes (W%)		10.5	15.4	13.9	4.9	31.7	17.6	7.6	18.3
No (ref, W%)		7.2	11.3	8.8	2.3	23.5	5.4	3.7	7.2
OR		1.51 (0.74–3.07)	1.43 (0.79–2.56)	1.67 (0.87–3.22)	2.12 (0.68–6.63)	1.51 (0.96–2.36)	**3.72 (1.95–7.1)**	2.17 (0.88–5.34)	**2.88 (1.55–5.37)**
History of CAN	330 (48.6)								
Yes (W%)		8.4	16.2	12.9	4.0	31.7	10.8	5.7	12.0
No (ref, W%)		7.0	8.0	6.6	1.6	18.4	4.5	3.1	6.4
OR		1.22 (0.64–2.27)	**2.21 (1.33–3.67)**	**2.09 (1.19–3.66)**	2.49 (0.78–7.99)	**2.06 (1.41–2.98)**	**2.59 (1.31–5.09)**	1.90 (0.82–4.41)	**2.0 (1.09–3.66)**
**Parental exposure to violence during adulthood**									
Physical/sexual IPV	225 (31.8)								
Yes (W%)		13.2	20	17.4	6.4	41.8	12.3	8.3	15.3
No (ref, W%)		5.1	8.1	6.0	1.0	16.8	5.3	2.5	6.2
OR		**2.83 (1.57–5.10)**	**2.81 (1.66–4.77)**	**3.31 (1.89–5.77)**	**6.4 (2.12–19.47)**	**3.56 (2.40–5.29)**	**2.50 (1.32–4.73)**	**3.58 (1.64–7.84)**	**2.74 (1.53–4.89)**
Non-partner physical/sexual violence	217 (30.3)								
Yes (W%)		7.1	15.1	16.2	6.3	31.1	12.0	6.3	13.2
No (ref, W%)		8.1	10.5	6.7	1.2	22.2	5.5	3.4	7.3
OR		0.87 (0.47–1.61)	1.50 (0.89–2.54)	**2.68 (1.58–4.54)**	**5.52 (2.01–15.11)**	**1.58 (1.08–2.33)**	**2.34 (1.24–4.45)**	1.89 (0.85–4.22)	**1.95 (1.08–3.51)**
Exposure to at least one type of violence during adulthood	347 (48.0)								
Yes (W%)		11.3	16.3	14.3	5.0	34.3	10.6	6.3	12.7
No (ref, W%)		4.4	7.9	5.3	0.7	15.9	4.6	2.5	5.8
OR		**2.77 (1.47–5.21)**	**2.26 (1.30–3.94)**	**2.96 (1.66–5.28)**	**7.55 (2.10–27.13)**	**2.76 (1.84–4.13)**	**2.47 (1.24–4.93)**	**2.56 (1.14–5.75)**	**2.37 (1.28–4.36)**
**Parental cumulative violence exposure**									
No report of violence (ref)	286 (41.7)	4.8	8.2	4.5	0.6	16.1	4.8	2.1	5.7
Only childhood (W%)	53 (9.6)	2.6	6.5	10.4	1.3	16	2.6	5.2	5.2
OR		0.53 (0.11–1.49)	0.78 (0.18–3.35)	2.45 (0.74–8.08)	2.24 (0.19–25.90)	0.99 (0.39–2.52)	0.53 (0.06–4.41)	2.5 (0.54–11.86)	0.90 (0.21–3.71)
Only adulthood (W%)	183 (25.4)	8.9	14.8	9.4	3.9	28.2	4.9	4.5	8.4
OR		1.93 (0.90–4.14)	1.94 (0.99–3.81)	**2.19 (1.07–4.48)**	**6.81 (1.28–36.3)**	**2.05 (1.26–3.33)**	1.03 (0.39–2.76)	2.18 (0.63–7.51)	1.52 (0.64–3.60)
Both (W%)	154 (23.2)	12.4	17.8	19.9	6.5	39.9	17.7	8.6	18.1
OR		**2.80 (1.38–5.68)**	**2.44 (1.26–4.70)**	**5.25(2.72–10.11)**	**11.51 (2.37–55.9)**	**3.45 (2.14–5.58)**	**4.27 (1.99–9.16)**	**4.37(1.69–11.26)**	**3.64 (1.79–7.42)**

*OR: unadjusted odds ratio with 95%CIs*.

*W% are weighted percentages*.

*Bold font indicates significant results at p < 0.05*.

**Table 3:** Parental violence exposure by socio-demographic characteristics.

	**Parental violence exposure during childhood**					**Parental violence exposure during adulthood**		
	**Psychologically abused as a child**	**Physically abused as a child**	**Sexually abused as a child**	**IPV witnessing**	**Exposure to at least one type of violence**	**Physical/sexual IPV**	**Non-partner physical/sexual violence**	**Exposure to at least one type of violence during adulthood**
Relationship with child								
Father (ref, W%)	27.9	16.6	10.0	12.7	40.6	29.9	40.0	54.4
Mother (W%)	31.0	19.3	24.8	17.7	48.6	30.9	16.3	38.7
OR	1.16 (0.96–1.40)	1.20 (0.96–1.50)	**2.99 (2.36–3.78)**	**1.48 (1.19–1.84)**	**1.38 (1.16–1.65)**	1.05 (0.86–1.28)	0.29 (0.23–0.36)	**0.53 (0.44–0.63)**
Ethnicity								
European (ref,W%)	27.6	15.8	15.7	12.07	42.7	30.6	29.3	47.2
Maori (W%)	53.4	33.7	29.6	32.6	71.3	45.5	35.0	60.0
OR	**3.00 (2.26–4.00)**	**2.71 (1.96–3.74)**	**2.25 (1.67–3.04)**	**3.52 (2.60–4.77)**	**3.33 (2.44–4.54)**	**1.89 (1.42–2.51)**	1.29 (0.97–1.73)	**1.67 (1.24–2.24)**
Pasifika (W%)	28.4	20.3	19.1	18.7	42.4	25.8	24.4	41.1
OR	1.04 (0.7–1.55)	1.36 (0.90–2.05)	1.26 (0.76–2.11)	**1.67 (1.11–2.50)**	0.99 (0.67–1.46)	0.78 (0.48–1.27)	0.78 (0.52–1.15)	0.78 (0.51–1.20)
Asian (W%)	19.2	12.9	12.9	11.6	32.8	20.2	20.7	35.4
OR	**0.62 (0.47–0.82)**	0.79 (0.55–1.13)	0.79 (0.56–1.12)	0.95 (0.65–1.39)	**0.65 (0.52–0.83)**	**0.57 (0.41–0.79)**	**0.63 (0.45–0.88)**	**0.61 (0.46–0.81)**
Food security								
Secure (ref, W%)	26.5	16.3	14.9	13.5	41.8	26.4	26.3	43.1
Insecure (W%)	42.2	25.2	27.5	23.0	56.8	47.3	36.3	60.4
OR	**2.02 (1.62–2.52)**	**1.73 (1.33–2.34)**	**2.17 (1.69–2.80)**	**1.92 (1.46–2.52)**	**1.83 (1.46–2.30)**	**2.49 (1.99–3.12)**	**1.59 (1.26–2.00)**	**2.01 (1.60–2.54)**

*OR: unadjusted odds ratio with 95%CIs*.

*W% are weighted percentages*.

*Bold font indicates significant results at p < 0.05*.

To understand the impact of each type of parental violence exposure during childhood and adulthood on their children's outcomes, a series of multivariable logistic regressions were conducted with exposure variables entered one by one into logistic regression analyses after adjusting for those socio-demographic characteristics that had a significant association with children's outcomes at the univariate level (e.g., parent's gender, ethnicity, and food security status). The results were presented as adjusted odds ratios (AOR) with 95% CIs (**Table 5**). We sought to control for the effect of socio-demographic characteristics because of the known association between child outcomes and socio-demographics characteristics.

A multivariable logistic regression was also conducted to investigate the impact of parental cumulative violence exposure adjusted for socio-demographic characteristics (**Table 5**). Finally, to determine whether there were any gender differences in the association between each parental exposure variable and child outcomes, multivariable logistic regression models were conducted with each exposure variable, gender and interaction terms (between each exposure and gender) included. These regression models were also adjusted for socio-demographic variables. As no significant interaction effects were found, the results of multivariable logistic regression in **Table 5** were not stratified by gender. However, due to the gender-based nature of violence discussed in literature (10, 11, 23) and to explore any gender differences which could not be captured by interaction terms, multivariable analyses stratified by gender are presented in Supplementary Tables 1–3.

## Results

### Study Sample Characteristics and Prevalence of Parental Exposure to Violence During Childhood and/or Adulthood

Mothers constituted half of the study sample (50.3%). Respondents ranged in age from 20 to 71 years, mean = 43.5, SD = 7.4 (fathers ranged in age from 24 to 71 years, mean = 45.1, SD = 7.4 and mothers ranged in age from 20 to 56 years, mean = 42, SD = 7.0). The majority of the study sample (95.4%) were aged over 30. Those who identified as European constituted 59.1% of the sample, Māori 14.7%, Pasifika 9.4%, and Asian 17.7%. Over one quarter of the sample (26%) were classified as food insecure (Table 2).

Almost half of the study sample (parents) reported at least one type of violence exposure during their childhood (48.6%). The same proportion (48%) reported exposure to at least one type of violence during adulthood. Psychological abuse during childhood was the most prevalent childhood violence exposure and was reported by almost one third of the study sample (34.4%), followed by child physical abuse reported by one-fifth (20.8%) of the sample. Sexual abuse during childhood was reported by 18.4% of parents (24.8 % of mothers; 10% of fathers) and IPV witnessing was reported by 17.4% of parents (17.7% of mother; 12.7% of fathers) (Table 3). Exposure to physical or sexual violence by either a partner or non-partner were both reported by almost 30% of parents. Regarding cumulative violence exposure, 41.7% of parents reported no exposure to violence during childhood or adulthood. Less than ten percent (9.6%) of parents reported exposure to violence only during childhood and one quarter (25.4%) reported exposure to violence only during adulthood. Those parents who reported exposure to violence during both childhood and adulthood constituted 23.2% of the study sample (Table 2).

Mothers were more likely to report exposure to violence during childhood and were less likely to report exposure to non-partner violence during adulthood compared with fathers. Māori respondents were more likely to report exposure to violence during childhood and/or adulthood. Those identified as food insecure were more likely to report exposure to violence during childhood and/or adulthood (Table 3). Exposure to violence during childhood (any type) was significantly associated with exposure to violence during adulthood (any type) with AORs ranging from 2.17 (95% CI: 1.37–3.44) (for exposure to physical abuse as a child and exposure to physical and/or sexual IPV) to 8.38 (95% CI: 4.87–14.40) (for exposure to sexual abuse as a child and exposure to at least one type of violence during adulthood) (Table 4). Supplementary Table 1 shows these associations for mothers and fathers separately.

**Table 4:** Multivariable analyses: association between parental violence exposure during childhood and during adulthood.

**Parental violence exposure during childhood**	**Parental violence exposure during adulthood**		
	**Physical/Sexual IPV AOR**	**Non-partner physical/sexual violence AOR**	**Exposure to at least one type of violence during adulthood AOR**
Psychologically abused as a child	**2.60 (1.75–3.87)**	**3.55 (2.35–5.36)**	**3.27 (2.11–5.06)**
Physically abused as a child	**2.17 (1.37–3.44)**	**4.55 (2.67–7.77)**	**3.50 (1.92–6.37)**
Sexually abused as a child	**5.23 (3.29–8.32)**	**4.56 (2.75–7.55)**	**8.38 (4.87–14.40)**
IPV witnessing	**2.19 (1.36–3.51)**	**2.63 (1.60–4.31)**	**2.78 (1.71–4.53)**
Exposure to at least one type of violence	**3.13 (2.13–4.58)**	**3.80 (2.57–5.62)**	**4.00 (2.77–5.78)**

*AOR: adjusted odds ratio (with 95%CIs) adjusted for parent's gender, ethnicity, and food security status*.

*Bold font indicates significant results at p < 0.05*.

### Prevalence and Pattern of Child Emotional-Behavioural Difficulties as Reported by Parents

The most prevalent emotional-behavioural difficulty reported by parents was of a child being timid or withdrawn (11.9 %), followed by a child being aggressive with other children or parents (9.6%). A child having nightmares was reported by 7.8% of parents and a child running away from home was reported by 2.8% of parents. In general, one quarter of parents reported that their children aged 5–17 had at least one emotional-behavioural difficulty (Table 2). Mothers were more likely to report their children had at least one emotional-behavioural difficulty compared with reports from fathers (OR 1.71, 95% CI: 1.16–2.53). Those who identified as Asian were less likely to report that their child had at least one emotional-behavioural difficulty compared with those who identified as European (OR 0.40, 95% CI 0.20–0.78). Other differences between ethnic groups were not significant. Those who identified as food insecure were more likely to report that their children had at least one emotional-behavioural difficulty (OR 2.59, 95% CI:1.35–4.96).

### Parental Exposure to Violence During Childhood and Adulthood and Their Reports of Their Children's Emotional-Behavioural Difficulties

Parents with a history of CAN were more likely to report that they had a child who was timid (AOR 2.11, 95%CI: 1.26–3.57) or aggressive (AOR 1.82, 95%CI: 1.03–3.22) compared with parents with no history of CAN. In general, parents with a history of CAN were more likely to report that they had a child with at least one emotional-behavioural difficulty (AOR 1.85, 95%CI: 1.26–2.72) (Table 5).

**Table 5:** Multivariable analyses: association between parental exposure to violence and child's emotional-behavioural and school difficulties.

	**Child's emotional-behavioural difficulties**					**Child's school difficulties**		
	**Nightmare AOR**	**Timid AOR**	**Aggressive AOR**	**Run away from home AOR**	**At least one child behavioural/emotional difficulty AOR**	**Being truant from school AOR**	**Being suspended from school AOR**	**At least one child school difficulty AOR**
**Parental exposure to violence during childhood**								
Psychologically abused as a child (ref = no)	1.06 (0.55–2.02)	1.55 (0.93–2.58)	1.27 (0.72–2.23)	2.32 (0.85–6.32)	1.42 (0.95–2.11)	**2.22(1.11–4.45)**	1.16 (0.44–3.06)	1.63(0.87–3.05)
Physically abused as a child (ref = no)	0.97 (0.47–2.0)	0.97 (0.52–1.79)	**2.04 (1.09–3.80)**	**5.34(1.90–15.0)**	1.22 (0.75–1.97)	**2.93(1.50–5.71)**	**3.70 (1.59–8.58)**	**2.43(1.29–4.58)**
Sexually abused as a child (ref = no)	1.54 (0.78–3.00)	1.85 (0.97–3.52)	**3.19 (1.78–5.70)**	1.46 (0.50–4.28)	**2.48 (1.52–4.03)**	**3.02(1.35–6.75)**	1.52 (0.47–4.87)	2.07(0.98–4.37)
IPV witnessing (ref = no)	1.32 (0.63–2.77)	1.41 (0.78–2.52)	1.39 (0.69–2.77)	2.14 (0.70–6.50)	1.32 (0.83–2.11)	**3.52(1.84–6.74)**	1.98 (0.76–5.11)	**2.68(1.42–5.06)**
History of CAN (ref = no)	1.10 (0.57–2.11)	**2.11 (1.26–3.57)**	**1.82 (1.03–3.22)**	2.40 (0.77–7.48)	**1.85 (1.26–2.72)**	**2.42(1.20–4.86)**	1.78 (0.72–4.41)	1.84 (0.99–3.41)
**Parental exposure to violence during adulthood**								
Physical/Sexual IPV (ref = no)	**2.58 (1.40–4.74)**	**2.41 (1.41–4.13)**	**2.91 (1.61–5.25)**	**5.99 (1.76–20.4)**	**3.21 (2.13–4.85)**	**2.12(1.06–4.25)**	**3.08 (1.30–7.32)**	**2.31(1.23–4.34)**
Non-Partner Physical/sexual Violence (ref = no)	1.05 (0.57–1.92)	1.38 (0.79–2.42)	**3.14 (1.75**–**5.65)**	**5.20 (1.92–14.1)**	**1.73 (1.14–2.64)**	**2.40(1.23–4.68)**	1.85 (0.80–4.31)	**2.01(1.09–3.70)**
History of exposure to violence during adulthood (ref = no)	**2.95 (1.57–5.55)**	**1.97 (1.11–3.48)**	**2.88 (1.58–5.25)**	**6.75 (1.77–25.8)**	**2.73 (1.77–4.21)**	**2.21(1.07–4.57)**	2.26 (0.99–5.15)	**2.11(1.11–4.01)**
**Parental cumulative violence exposure**								
No report of violence	Ref	Ref	Ref	Ref	Ref	Ref	Ref	Ref
Only childhood abuse	0.43 (0.09–2.06)	0.77 (0.18–3.24)	2.19 (0.66–7.31)	2.40 (0.21–27.6)	0.87 (0.34–2.23)	0.52(0.06–4.57)	2.47 (0.47–12.9)	0.85 (0.19–3.7)
Only adulthood abuse	**2.45 (1.14–5.30)**	1.96 (0.98–3.93)	**2.47 (1.21–5.05)**	**6.47 (1.22–34.2)**	**2.33 (1.40–3.90)**	1.06(0.41–2.73)	2.25 (0.68–7.45)	1.62 (0.69–3.8)
Both	**2.60 (1.27–5.35)**	**2.43 (1.27–4.64)**	**5.03 (2.62–9.65)**	**11.9(2.40–58.4)**	**3.32 (2.05–5.38)**	**4.22(1.94–9.21)**	**4.30 (1.59–11.6)**	**3.56 (1.73–7.3)**

*AOR: adjusted odds ratio (with 95%CIs) adjusted for parent's gender, ethnicity and food security status*.

*Bold font indicates significant results at p < 0.05*.

Parents exposed to IPV were more likely to report that they had a child with any type of emotional-behavioural difficulty, with AORs ranging from 2.41 (95% CI: 1.41–4.13) for a child being timid to 5.99 (95%CI: 1.76–20.37) for a child running away from home. Parents with exposure to non-partner violence during adulthood were more likely to report that they had a child who had been aggressive (AOR 3.14, 95% CI: 1.75–5.65) or run away from home (AOR 5.20, 95% CI: 1.92–14.1). Parents with any exposure to violence during adulthood (by an intimate partner or non-partner) were more likely to report that they had a child with any type of emotional-behavioural difficulty, with AORs ranging from 1.97 (95%CI: 1.11–3.48) for being timid to 6.75 (95%CI: 1.77–25.78) for running away from home. Parents who had been exposed to violence during adulthood had increased odds of reporting that they had a child with at least one emotional-behavioural problem (AOR 2.73, 95%CI: 1.77–4.21) (Table 5).

Parents with no history of abuse during childhood and/or adulthood had lower rates of reporting that their children experienced emotional-behavioural difficulties (Table 2). Parents who had been exposed to only violence in childhood did not report that their children had more emotional-behavioural difficulties compared to parents without any history of violence exposure during childhood and adulthood. However, parents with violence exposure only during adulthood had higher odds of reporting emotional-behavioural problems for their children, ranging from 1.96 (95% CI: 0.98–3.93) for being timid to 6.47 (95% CI: 1.22–34.22) for running away from home after adjusting for socio-demographic factors. Similarly, parents who reported that they had a history of violence exposure during both childhood and adulthood had higher odds of reporting that they had a child with emotional-behavioural difficulties compared with parents with no history of violence exposure (Table 5). The results of multivariable analyses stratified by parent's gender are presented in Supplementary Tables 2, 3. The associations show similar patterns for mothers and fathers, however the associations were stronger for mothers.

### Prevalence and Pattern of Parental Reports of Child School Difficulties

Having a child who was truant from school was reported by 7.5 % of parents. Having a child who was suspended from school was less common and was reported by 4.3% of parents. In general, 9.1% of parents reported that they had a child with at least one school difficulty. Those who identified as Māori or Pasifika were more likely to report that their children had at least one school difficulty (OR 2.33, 95% CI: 1.07–5.09 for Māori; OR 2.77, 95% CI: 1.21–6.35 for Pasifika) compared with those who identified as European. Those who identified as Asian were less likely to report at least one school-related problem compared with those who identified as European (OR 0.17, 95% CI: 0.04–0.74) (Table 2). Those who identified as food insecure were more likely to report at least one school difficulty compared with those who identified as food secure (OR 2.63, 95% CI: 1.45–4.77). No significant association was found between gender of parent and child school difficulties.

### Parental Violence Exposure During Childhood and Adulthood and Their Reports of Their Children's School Difficulties

Parents who were exposed to any type of violence during childhood (psychological, physical, or sexual abuse or IPV witnessing) were more likely to report that they had a child who had been truant from school (ranging from AOR 2.22, 95% CI: 1.11–4.45 for parents with exposure to child psychological abuse to AOR 3.53, 95% CI: 1.84–6.74 for parents who had witnessed IPV as a child). Parents who were physically abused during childhood were also more likely to report that they had a child who had been suspended from school (AOR 3.70, 95% CI: 1.59–8.58) (Table 5).

Parents who had been exposed to any type of violence during adulthood (by an intimate partner or non-partner) were also more likely to report that they had a child who had been truant from school (range: AOR 2.12, 95% CI: 1.06–4.25 for parents exposed to physical and/or sexual IPV to AOR 2.40, 95% CI: 1.23–4.68 for parents exposed to non-partner physical and/or sexual violence). Parents who reported exposure to physical/sexual IPV were also more likely to report that they had a child who had been suspended from school (AOR 3.08, 95% CI: 1.30–7.32) (Table 5).

Only parents who reported that they had been exposed to both violence during childhood and violence during adulthood reported that their children had more school difficulties compared with parents with no history of violence exposure during childhood and adulthood (AOR 3.56, 95% CI: 1.73–7.31 for at least one child school difficulty) (Table 5). The results of multivariable analyses stratified by parent's gender are presented in Supplementary Tables 2, 3. As with emotional-behavioural difficulties, the associations show similar patterns for mothers and fathers, however the associations were stronger for mothers.

## Discussion

In this New Zealand cohort of parents of children aged 5–17 years old, parental exposure to violence during childhood and adulthood was prevalent. Almost half of the study sample (parents) (48.6%) retrospectively reported exposure to violence during their childhood and the same proportion (48%) reported exposure to violence during their adulthood life. Parental exposure to violence during childhood and adulthood was more likely to be reported by those who were identified as Māori and those who identified as food insecure. Ethnic differences might be due to experiences of colonisation, and historical and cumulative trauma (24). Mothers were more likely to report exposure to sexual abuse as a child and to report having witnessed IPV against their mother/stepmother.

Parents who reported exposure to violence during childhood were also more likely to report exposure to violence during adulthood (experience of both IPV and non-partner violence). Specifically, parents who reported exposure to at least one type of violence during childhood were four times more likely to report exposure to at least one type of violence during adulthood. This finding is consistent with evidence indicating that children who have been exposed to maltreatment are at increased risk of continued maltreatment by others (25–27). The findings also support evidence from smaller studies, primarily conducted with shelter or refuge samples (28) showing that women who experience physical or sexual abuse in childhood were more likely to report adult experiences of IPV.

In general, children of parents with histories of exposure to violence during childhood were at increased risk for experiencing emotional-behavioural or school difficulties. These findings are consistent with previous studies (5, 9). However, where parents reported a history of childhood abuse but not adult experience of violence, their children had similar odds of experiencing difficulties to the children of parents who had not been exposed to any violence in their lifetime. Such results highlight an opportunity for effective early intervention to limit or ameliorate the impact of violence exposure during childhood by preventing experience of violence in adulthood and to ultimately break the intergenerational cycle of violence and disadvantage. Suggestions for way to interrupt these cycles of violence come from work on fostering resilience, which outlines ways in which individuals can be supported to develop a “density and diversity of assets and resources” that can help them overcome violence exposure. These suggestions include helping individuals to build strengths, such as the development of emotional regulation, and interpersonal and meaning-making skills. It also includes assisting people to develop resources and assets through providing opportunities to connect with supportive environments, such as with nurturing schools and community organisations. Enhancing networks that support cultural connexion and strength can also foster the development of resilience (24, 29–31). Development and implementation of these supportive cultural strategies may be especially important as our research, and other research, indicates that Māori students are more likely to be stood down, suspended, and excluded from schools than any other ethnic group (32).

Children of parents who had been exposed to violence only during adulthood were at higher risk of experiencing emotional-behavioural difficulties compared with children of parents with no violence exposure. This is consistent with other studies which have reported that experience of violence in intimate relationships is associated with poorer outcomes for their children (8, 9, 26, 33). It is reasonable to postulate that the abusing partner might undermine their partner's parenting ability, which in turn could hinder the development of effective child-parent relationships and functioning of their children (5, 9, 26). Strategies to resolve such difficulties include identifying who is the non-abusing parent and supporting them to be a safe, secure attachment for the child(ren). It is also likely that children who live in homes where IPV occurs are more likely to be directly abused and neglected (27, 34) which increases their likelihood of experiencing difficulties at home and/or at school (27). Both of these points highlight the importance of developing strategies that contain, challenge and change the behaviour of the person using violence (14), and that work to resolve these abusive behaviours before seeking to reestablish a relationship between the parent and child (35, 36).

Children of parents with histories of exposure to violence during both childhood and adulthood had the highest prevalence of experiencing emotional/behavioural and school difficulties. This indicates that cumulative violence exposure throughout the lifespan is associated with poorer outcomes compared with exposures to violence at a single point in life. A possible explanation for this finding could be that those with cumulative violence exposure are more likely to suffer from serious mental health conditions such as depression, anxiety, and complex PTSD which in turn would have an impact on their child rearing (9, 37). Indeed, long-lasting relational effects of exposure to violence during childhood such as a range of physical and psychological morbidities, exacerbated by cumulative traumatic experiences as an adult can impede the capacity of parents to nurture and care for children, leading to ‘intergenerational cycles’ of trauma (38).

Parents with a history of violence are also more likely to have multiple socio-economic challenges, including unintended pregnancies (39), antenatal and postnatal depression (40), contact with the justice system and low employment (41) which can also preclude the capacity of parents to nurture and care for children (38). Our findings support other studies reporting that experience of multi forms of violence is associated with greater adverse impacts (9, 25, 37). These findings highlight the intergenerational impacts of family violence and the complex intersections between parents' and children's life experiences. However, it is important to note that many children of parents with previous exposure to violence (either during childhood or adulthood or both) were not identified as having emotional-behavioural and/or school difficulties. It is possible that some parents may under-report difficulties that children are experiencing, but equally possible that some families, schools and health services are effectively supporting these children. Resilience research has shown that family support and extra-family links, school and peer support have all been correlated with greater resiliency and better functioning in children exposed to violence (42, 43). Research utilising Māori and Indigenous knowledge has also demonstrated how these approaches can strengthen whānau (family) resilience (44, 45).

### Implications

For those children who are experiencing emotional-behavioural and school difficulties, these findings highlight the importance of screening for violence exposure with the children and with the children's families (e.g., in before school checks to identify children's needs). This would support the identification of those most in need of intervention and provide the opportunity to deliver supportive strategies and guided referrals as indicated. Early screening may bring us closer to identifying youth at an earlier phase in their lifespan, which could provide opportunities to mitigate the effects of violence exposure and reduce their risk of subsequent victimisation. Enhancing positive, supportive relationships between parents and children and between parents and other adults could be a key prevention strategy for interrupting the cycle of child maltreatment. This in turn would have benefits for subsequent generations.

### Strengths

The strengths of this study include the large general population cohort, the assessment of multiple violence exposures over the lifespan using standardised measures, inclusion of exposure to both partner and non-partner violence during adulthood, assessment of a wide range of emotional-behavioural and school difficulties among children, and inclusion of both mothers and fathers.

### Limitations

Using parental report of child outcomes may introduce bias. This may come from many sources, including parental cultural knowledge and perceptions of child behaviours, or parent's feelings of shame or stigma. Use of multiple informants, e.g., teachers, would be beneficial to validate parent's reports of children's difficulties. Due to these limitations, it is likely that our prevalence estimates underreport the extent of child difficulties. It is also plausible that we may have missed the most serious cases as individuals with severe problems are less likely to participate in population-based studies like this. No casualty can be inferred. Further research is needed to understand the mechanisms through which parental exposure to violence results in children's difficulties, and if this exposure has a differential effect for boys vs. girls. More importantly, further research on factors that mitigate the negative impacts of parental exposure to violence on their children's outcomes is urgently required. Future studies could expand this work by exploring the impacts of other parental adversities on their children's well-being. Inclusion of data on the violence exposure of both parents (the parental dyad) would also assist in exploring if there are cumulative effects of this exposure on children.

## Conclusion

Our findings indicate that children are at risk of experiencing difficulties when their parents have been exposed to violence. These results suggest the need for expanded prevention services and parent support for those already exposed to the violence. Our findings indicate that difficulties experienced by a considerable number of young children could be reduced by stopping violence. The accumulation of risk within families (child abuse, IPV, non-partner violence) highlights the need for effective early intervention to limit or ameliorate the impact of violence across the lifespan, to build resilience and foster the development of safe, stable and nurturing relationships in order to break the inter-generational cycle ofdisadvantage.

Better support of parents with a history of violence in childhood, and/or adulthood has the potential to disrupt the intergenerational experiences of violence and profoundly influence the lives of children and families. Strengthening the capacity of mental health and school professionals to recognise and respond to family violence and building stronger evidence about effective and timely interventions involving the health and education sectors are critical priorities for safeguarding the health of future generations.

## Data Availability Statement

The datasets presented in this article are not readily available because the confidentiality and sensitivity of the data and Māori data sovereignty. Requests to access the datasets should be directed to j.fanslow@auckland.ac.nz.

## Ethics Statement

The study was reviewed by the University of Auckland Human Participants Ethics Committee (reference number 2015/018244). The participants provided their written informed consent to participate in the study. The patients/participants provided their written informed consent to participate in this study.

## Author Contributions

LH organized the database, performed the statistical analysis, and wrote the first draft of the manuscript. JF contributed to conceptualization, funding acquisition, project administration, supervision of the study, and writing—review and editing. PG contributed to the conceptualization, funding acquisition, and writing-review and editing. TM contributed to the funding acquisition and writing-review and editing. All authors contributed to manuscript revision, read, and approved the submitted version.

## Funding

This study received funding from the New Zealand Ministry of Business, Innovation and Employment, Contract number CONT-42799-HASTR-UOA.

## Conflict of Interest

The authors declare that the research was conducted in the absence of any commercial or financial relationships that could be construed as a potential conflict of interest.

## Publisher's Note

All claims expressed in this article are solely those of the authors and do not necessarily represent those of their affiliated organizations, or those of the publisher, the editors and the reviewers. Any product that may be evaluated in this article, or claim that may be made by its manufacturer, is not guaranteed or endorsed by the publisher.
