# Supplementary material for: Intergenerational Impact of Violence Exposure: Emotional-Behavioural and School Difficulties in Children Aged 5–17
Source: Front Psychiatry. 2022 Jan 4;12:771834. doi: 10.3389/fpsyt.2021.771834 (PMC8764379; doi:10.3389/fpsyt.2021.771834)
Supplement: Supplementary file 1 [file Table_1.DOCX]

**Supplementary Table 1. Multivariable analyses: association between parental violence exposure during childhood and adulthood by parental gender**

|  | **Maternal exposure to violence during adulthood** | | | **Paternal exposure to violence during adulthood** | | |
| --- | --- | --- | --- | --- | --- | --- |
|  | **Physical/Sexual IPV**  **AOR** | **Non-Partner Physical/sexual Violence**  **AOR** | **Exposure to at least one type of violence during adulthood**  **AOR** | **Physical/Sexual IPV**  **AOR** | **Non-Partner Physical/sexual Violence**  **AOR** | **Exposure to at least one type of violence during adulthood**  **AOR** |
| **Psychologically abused as a child** | **2.34 (1.35-4.05)** | **5.31 (2.76-10.21)** | **3.51 (1.91-6.46)** | **3.15 (1.78-5.55)** | **2.70 (1.57-4.64)** | **3.41 (1.85-6.30)** |
| **Physically abused as a child** | **2.14 (1.11-4.12)** | **7.75 (3.67-16.34)** | **3.59 (1.53-8.46)** | **2.25 (1.20-4.21)** | **2.78 (1.38-5.59)** | **3.62 (1.57-8.37)** |
| **Sexually abused as a child** | **6.04 (3.51-10.39)** | **4.83 (2.73-8.56)** | **8.68 (4.79-15.74)** | **3.36 (1.41-8.02)** | **3.44 (1.38-8.56)** | **7.79 (2.04-29.78)** |
| **IPV witnessing** | **2.94 (1.58-5.51)** | **3.90 (2.06-7.36)** | **3.53 (1.87-6.64)** | 1.54 (0.72-3.25) | 1.66 (0.80-3.45) | **2.19 (1.03-4.66)** |
| **Exposure to at least one type of violence** | **3.92 (2.29-6.73)** | **7.18 (3.39-15.21)** | **6.00 (3.50-10.28)** | **2.70 (1.58-4.62)** | **2.83 (1.73-4.64)** | **3.16 (1.91-5.21)** |

AOR: adjusted odds ratio (with 95%CIs) adjusted for ethnicity and food security status

Bold font indicates significant results at p < 0.05.

**Supplementary Table 2. Multivariable analyses: association between maternal** **violence exposure and child’s emotional-behavioural and school difficulties, the 2019 New Zealand Family Violence Study**

|  | Child’s emotional-behavioural difficulties | | | | | Child’s school difficulties | | |
| --- | --- | --- | --- | --- | --- | --- | --- | --- |
|  | Nightmare  AOR | Timid  AOR | Aggressive  AOR | Run away from home  AOR | At least one child behavioral/emotional difficulty AOR | Being truant from school  AOR | Being suspended from school  AOR | At least one child school difficulty  AOR |
| **Mother’s violence exposure during childhood** | | | | | | | | |
| Psychologically abused as a child (ref=no) | 0.80(0.36-1.76) | 0.94 (0.48-1.84) | 1.54 (0.75-3.15) | 1.78 (0.46-6.79) | 1.06 (0.63-1.79) | 1.85 (0.73-4.66) | 0.65 (0.18-2.32) | 1.40 (0.62-3.13) |
| Physically abused as a child (ref=no) | 0.63(0.25-1.61) | 0.85 (0.35-2.09) | 1.97 (0.84-4.60) | **9.2 (2.09-40.49)** | 1.02 (0.53-1.98) | **2.93 (1.28-6.72**) | 2.12 (0.75-6.03) | **2.31 (1.06-5.04)** |
| Sexually abused as a child (ref=no) | 1.54(0.75-3.19) | **2.18 (1.07-4.46)** | **4.50 (2.22-9.11)** | 2.28 (0.61-8.58) | **3.07 (1.79-5.26)** | **2.54 (1.05-6.15)** | 0.97 (0.28-3.38) | 1.68 (0.76-3.72) |
| IPV witnessing (ref=no) | 1.17(0.50-2.74) | 1.36 (0.66-2.75) | 1.44 (0.59-3.51) | 2.59 (0.57-11.8) | 1.45 (0.80-2.62) | **5.41(2.31-12.7)** | 2.01 (0.64-6.26) | **3.74 (1.69-8.28)** |
| History of CAN (ref=no) | 0.83(0.38-1.80) | **2.22 (1.05-4.70)** | **2.70 (1.23-5.94)** | 3.62 (0.75-17.5) | **1.76 (1.03-3.01)** | 2.03 (0.83-4.96) | 1.31 (0.48-3.55) | 1.58 (0.74-3.36) |
| **Mother ’s violence exposure during adulthood** | | | | | | | | |
| Physical/Sexual IPV (ref=no) | **3.32(1.61-6.80)** | **2.64 (1.37-5.10)** | **3.07 (1.38-6.82)** | **10.30(1.81-58.5)** | **3.82 (2.19-6.68)** | 2.36 (0.92-6.05) | **2.69 (1.03-7.05)** | **2.28 (0.99-5.22)** |
| Non-Partner Physical/sexual Violence (ref=no) | 0.81(0.34-1.93) | 1.41 (0.64-3.11) | **3.89 (1.85-8.18)** | 2.80 (0.58-13.7) | **2.04 (1.13-3.67)** | 2.11 (0.87-5.10) | 1.16 (0.31-4.37) | 1.42 (0.60-3.35) |
| History of exposure to violence during adulthood (ref=no) | **3.07(1.44-6.51)** | **2.67 (1.26-5.64)** | **3.45 (1.56-7.63)** | **6.41 (1.14-36.0)** | **3.81 (2.20-6.61)** | 1.85 (0.71-4.86) | 1.62 (0.62-4.20) | 1.66 (0.72-3.84) |
| **Mother’s cumulative violence exposure** | | | | | | | | |
| No report of violence | Ref | Ref | Ref | Ref | Ref | Ref | Ref | Ref |
| Only childhood abuse | 0.42(0.08-2.15) | 1.34 (0.22-7.97) | 2.03 (0.42-9.67) | 2.94 (0.17-51.30) | 0.85 (0.26-2.76) | No observation | 0.53 (0.06-4.88) | 0.23 (0.02-1.92) |
| Only adulthood abuse | 2.49(0.92-6.70) | 2.66 (0.90-7.82) | 1.62 (0.48-5.41) | 6.28 (0.47-83.67) | **2.83 (1.33-6.00)** | 0.18 (0.01-1.76) | 1.44 (0.37-5.65) | 0.71 (0.18-2.75) |
| Both | **2.36(1.02-5.43)** | **3.42 (1.43-8.21)** | **5.60(2.36-13.32)** | **10.3(1.14-92.8)** | **4.18 (2.27-7.71)** | **2.67 (1.09-6.53)** | 1.49 (0.52-4.27) | 2.10 (0.92-4.79) |

AOR: adjusted odds ratio (with 95%CIs ) adjusted for mother’s ethnicity and food security status

Bold font indicates significant results at p < 0.05.

**Supplementary Table 3. Multivariable analyses: association between paternal violence exposure and child’s emotional-behavioural and school difficulties, the 2019 New Zealand Family Violence Study**

|  | Child’s emotional-behavioural difficulties | | | | | Child’s school difficulties | | |
| --- | --- | --- | --- | --- | --- | --- | --- | --- |
|  | Nightmare  AOR | Timid  AOR | Aggressive  AOR | Run away from home  AOR | At least one child behavioral/emotional difficulty AOR | Being truant from school  AOR | Being suspended from school  AOR | At least one child school difficulty  AOR |
| **Fathers’ violence exposure during childhood** | | | | | | | | |
| Psychologically abused as a child (ref=no) | 1.90 (0.61-5.87) | **2.50 (1.19-5.26)** | 0.94 (0.38-2.34) | 2.81 (0.67-11.8) | **2.08 (1.15-3.76)** | **2.76 (0.99-7.71)** | 2.25 (0.52-9.64) | 2.05 (0.80-5.26) |
| Physically abused as a child (ref=no) | 2.05 (0.69-6.08) | 1.08 (0.45-2.59) | 2.08 (0.84-5.18) | 3.42 (0.80-14.5) | 1.50 (0.74-3.03) | 2.83 (0.94-8.48) | **7.16(1.53-33.41)** | 2.59 (0.91-7.36) |
| Sexually abused as a child (ref=no) | 1.86 (0.36-9.51) | 1.26 (0.29-5.53) | 0.48 (0.60-3.86) | No observation | 1.01 (0.25-3.98) | **4.25 (1.0-18.15)** | 4.14(0.66-25.97) | 3.48 (0.85-14.27) |
| IPV witnessing (ref=no) | 1.93 (0.54-6.83) | 1.46 (0.54-3.92) | 1.41 (0.50-4.00) | 1.94 (0.37-10.3) | 1.13 (0.51-2.52) | **1.79 (0.52-6.10)** | 1.97(0.38-10.10) | 1.49 (0.45-4.95) |
| History of CAN (ref=no) | 2.06 (0.67-6.33) | **1.99 (0.95-4.11)** | 1.08 (0.48-2.40) | 1.80 (0.41-7.85) | **1.93 (1.11-3.37)** | **2.93 (1.00-8.58)** | 2.60(0.52-13.02) | 2.24 (0.82-6.17) |
| **Father’s violence exposure during adulthood** | | | | | | | | |
| Physical/Sexual IPV (ref=no) | 1.70 (0.60-4.80) | **2.42 (1.10-5.28)** | **2.62 (1.10-6.20)** | 4.51 (0.92-22.19) | **2.72 (1.50-4.92)** | 1.96 (0.66-5.80) | 3.84(0.85-17.31) | 2.38 (0.88-6.41) |
| Non-Partner Physical/sexual Violence (ref=no) | 1.49 (0.56-3.97) | 1.37 (0.63-2.95) | 2.11 (0.92-4.81) | **15.4 (1.7-139.8)** | 1.45 (0.81-2.59) | 2.79 (0.95-8.16) | 3.09 (0.76-12.6) | **3.05 (1.12-8.29)** |
| History of exposure to violence during adulthood (ref=no) | **3.90 (1.03-14.7)** | 1.65 (0.74-3.66) | 2.09 (0.82-5.33) | **9.77 (1.10-86.9)** | **1.88 (1.00-3.55)** | 3.21 (0.82-12.5) | 4.12(0.67-25.12) | 3.17 (0.95-10.55) |
| **Father’s cumulative violence exposure** | | | | | | | | |
| No report of violence | Ref | Ref | Ref | Ref | Ref | Ref | Ref | Ref |
| Only childhood abuse | No observation | No observation | 2.77 (0.28-27.2) | No observation | 0.67 (0.07-6.10) | 4.93 (0.33-73.5) | 1.98 (0.26-14.6) | 7.38 (0.70-77.4) |
| Only adulthood abuse | 2.75 (0.59-12.7) | 1.36 (0.58-3.18) | 2.41 (0.91-6.38) | 6.90 (0.71-66.5) | 1.63 (0.82-3.24) | 2.88 (0.55-15.0) | 0.37 (0.8-1.78) | 3.92 (0.77-19.9) |
| Both | **4.71 (1.21-18.22)** | 1.47 (0.54-4.0) | 2.69 (0.90-7.95) | **12.12(1.2-121.1)** | 2.04 (0.91-4.55) | 7.10 (1.33-37.8) | No observation | **7.12 (1.36-37.3)** |

AOR: adjusted odds ratio (with 95%CIs ) adjusted for father’s ethnicity and food security status

Bold font indicates significant results at p < 0.05.
